# Horizontal Violence and the Quality and Safety of Patient Care: A Conceptual Model

**DOI:** 10.1155/2012/306948

**Published:** 2012-05-09

**Authors:** Christina Purpora, Mary A. Blegen

**Affiliations:** ^1^School of Nursing and Health Professions, University of San Francisco, 2130 Fulton Street, San Francisco, CA 94117, USA; ^2^Department of Community Health Systems, School of Nursing, University of California, San Francisco, 2 Koret Way, P.O. Box 0608, San Francisco, CA 94143, USA

## Abstract

For many years, nurses in international clinical and academic settings have voiced concern about horizontal violence among nurses and its consequences. However, no known framework exists to guide research on the topic to explain these consequences. This paper presents a conceptual model that was developed from four theories to illustrate how the quality and safety of patient care could be affected by horizontal violence. Research is needed to validate the new model and to gather empirical evidence of the consequences of horizontal violence on which to base recommendations for future research, education, and practice.

## 1. Introduction

For several decades, clinical and academic nurses have written about horizontal violence among nurses in clinical settings and its consequences. Horizontal violence is behavior that is directed by one peer toward another that harms, disrespects, and devalues the worth of the recipient while denying them their basic human rights [1]. Examples include nonverbal behavior, such as ignoring a peer, verbal behavior, such as making sarcastic comments to them or talking behind their back, and/or physical acts like shoving someone or slamming things [1]. Other similar terms used to label negative behavior among nurses at work include nurse-on-nurse aggression [2, 3], bullying [4–8], verbal abuse [9–11], lateral violence [12–14], incivility [15], and lateral or horizontal hostility [16, 17]. The term horizontal violence was used in this paper because, unlike the other terms, horizontal violence is drawn from oppression theory, one of the four theories used to develop the model described herein. Research articles [2, 3, 5–7, 18, 19] and opinion pieces [20–22] from Australia, New Zealand, the United Kingdom, and the United States suggest that nurses share an ongoing and growing concern about horizontal violence and its consequences for nurses, nursing, healthcare organizations, and particularly for patients. 

Many researchers have described horizontal violence among nurses working in hospitals [2, 3, 5, 6, 10, 11, 14, 18, 23, 24]. Nurses suffer consequences as a result of their experiences such as sadness, anxiety, mistrust, diminished self-esteem and self-confidence [7, 10, 18], job dissatisfaction [10], and negative effects on peer relationships [10]. Some describe their experiences as painful [24] and far more distressing than when similar behaviors are directed toward them by physicians or patients [2, 3]. Some nurses intend to leave their current job to find work elsewhere [5, 6, 8, 11] while others consider leaving nursing altogether [5, 18]. Some nurses believe that horizontal violence threatens the safety of patients [18] and diminishes the quality of their care [10].

When behavior similar to horizontal violence occurs among healthcare providers from different disciplines, the term disruptive behavior is often used to name the behavior. Rosenstein and O'Daniel [25, 26] reported that doctors and nurses in hospitals perceive that disruptive behavior, such as use of rude tone of voice or threatening body language, decreases their communication. Communication decreases when individuals feel too intimidated to communicate with members of the healthcare team who are known instigators of these negative behaviors [26, 27]. The Joint Commission [28] reports that 60% of actual or potential harm to patients can be linked to insufficient communication in healthcare organizations. Yet, no direct empirical links among horizontal violence or disruptive behavior, communication, and patient care have been made. The dearth of research about the impact of horizontal violence on nurses' relationships and communication with each other and the concern about consequences for patients in the presence of horizontal violence call for studies of horizontal violence among nurses in hospitals, its effect on their relationships and communication, and the consequences for patient care.

To date, some researchers who study horizontal violence among nurses used Freire's [29] theory of oppression as a framework. Those who used it did so implicitly by using the term horizontal violence, one of its concepts [18, 30], while others did so explicitly [6, 14, 23, 24]. Conceptual models are important because of their utility for explaining situations and for guiding research [31], yet, none of the studies proposed a model to explain horizontal violence and its consequences for patient care.

This paper presents a conceptual model that illustrates how the quality and safety of patient care could be affected by horizontal violence. The paper begins with a description of the model in which Freire's oppression theory [29], Maslow's theory of human motivation [32], DeVito's essential human communication model [33], and Reason's Swiss cheese model of system accidents [34] are linked. Then, implications for research are provided.

## 2. Conceptual Model for Horizontal Violence and the Quality and Safety of Patient Care

**Figure 1:**
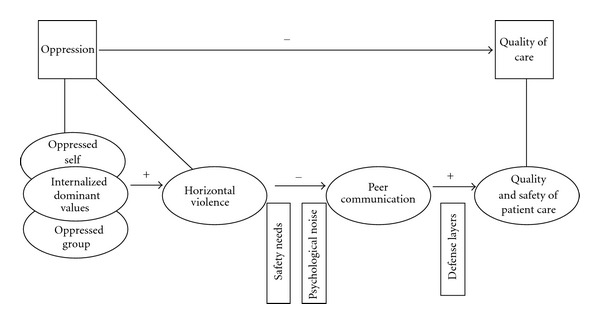
A conceptual model for horizontal violence and the quality and safety of patient care.

A conceptual model is an illustration of proposed causal relationships among a group of variables hypothesized to be associated with a problem [35]. The proposed horizontal violence and the quality and safety of patient care model displayed elsewhere [36] are shown in Figure 1. Directionality of the model flows from left to right.

### 2.1. Oppression

In his theory of oppression, Freire postulated that the Brazilian people he observed were living in a “situation of oppression” ([29, page 55]). They were dominated by others who had violently obstructed them from living their lives freely as human beings ensconced in their unique beliefs and values. Freire [29] contends that a situation of oppression can be changed because it results from an imbalanced social structure, not fate.

Building on the work of Freire and others, Roberts [37] posited that nurses have worked in a situation of oppression since the early 1900s when they began caring for patients in hospitals controlled by male physicians and administrators. Ashley [38] and Reverby [39] describe nurses in the mid 1800s to early 1900s doing the work traditionally thought of as the work of women in hierarchical hospitals. Their practice was controlled either by groups with more power that are held in higher esteem or by the systems in which they work. Power is defined as “… the ability to influence the decision and actions of others” ([40, page 38]). Today, nurses continue to bear a great deal of responsibility caring for patients whose lives are in their hands; yet they have little power compared to physicians and administrators [41].

### 2.2. Internalized Dominant Values and Horizontal Violence

Freire [29] theorized that oppressed people internalize their situation by adopting the dominant group's beliefs and values while minimizing their own. Oppressed people manifest what they internalize by acting like those who oppress them while remaining submissive to them. As the oppressed align with the oppressor, they develop hatred for their own group. This hatred is manifested when the oppressed themselves become oppressors of their group. The oppressed become fearful of fighting for freedom at the risk of more violence from those who oppress them who are threatened by the oppressed's struggle to break free from oppression [29]. Freire further postulates that the oppressed suffer from duality; they yearn for freedom and yet fear it. Duality splits the group, preventing them from engaging in a struggle for freedom.

Roberts [37] suggested that nurses have internalized the dominant physician values while minimizing those of nursing. She supports her argument by pointing to the prominence and value placed on the medical model over nursing. She further postulates that oppressed nurses manifest what they have internalized by exhibiting poor self-esteem, feelings of inferiority, aversion for nurses who are most often, but not always, women, dissatisfaction with the primarily female profession, disunity, and lack of professional identity. 

 Working with Roberts and others, DeMarco et al. [42] used the concepts “oppressed self” and “oppressed group” to explain how nurses' exhibit internalized dominant values while minimizing their own ([42, page 299]). Oppressed self demonstrates a person's beliefs about their individual worth. When people minimize their own worth, they may stay quiet rather than contribute their opinion in situations. Oppressed group represents beliefs about women, most nurses in hierarchical hospitals are women, and how they may be inclined to act together. When beliefs are negative, their collective contribution as women or nurses is minimized.

Freire used the term “horizontal violence” ([29, page 62]) to name a behavior he observed among oppressed Brazilians and a behavior first described by Fanon's [43] observation of oppressed Algerians. The concept was originally defined as acts of violence such as killing, burning each other's houses, and pulling knives on one another. Freire postulated that the oppressed feel aggressive but remain submissive toward those who oppress them and these violent acts occur as one way that oppressed people relieve mounting situational tension among them. Blanton and colleagues [1] used Freire's [29] work as well as others to develop the definition of horizontal violence used in the model proposed in this paper, the only one known to be derived from Freire's theory. Though the acts described by Blanton et al. between coworkers are not the same acts of horizontal violence defined by Freire, the concept is useful, nonetheless, for explaining behavior among nurses who are also thought to be oppressed.

In the model, horizontal violence represents the harmful behavior oppressed nurses are at risk for engaging in to relieve mounting frustration from working in hierarchical hospitals where they have great responsibility but little power. While there are many factors that influence nurses' work-related behavior, oppression is central and understudied. Other factors could include age, education, and experience. An assumption that nurses may engage in horizontal violence because they are oppressed has persisted in the nursing literature for at least three decades [6, 9, 24, 37]. The purpose of using the concepts of oppression, oppressed group, oppressed self, and horizontal violence in the proposed model is not to fault nurses [42, 44] but instead to explain, theoretically, why, as a group, nurses may be considered oppressed and, thus, at risk for engaging in horizontal violence. The proposition in the proposed model is that internalized dominant values are positively related to experiences of horizontal violence, that is, as internalized dominant values when exhibited as oppressed group or oppressed self increase, so does horizontal violence.

### 2.3. Horizontal Violence and Peer Communication

In his theory of human motivation, Maslow [32] explained how adult human behavior is motivated by several basic needs. Safety needs are centered on a human being's need to be free from physical and emotional harm. When a person's safety needs are met, they feel safe enough to relate to others. Conversely, a person who perceives the world as unsafe may believe their physical and emotional well-being are at risk for harm and may react to this threat by not relating to others. The concept is useful for explaining how nurses who have suffered psychological harm from horizontal violence may perceive threats to their emotional safety in work environments. Their hesitation or resistance to interacting with others may be in response to perceived threats to their emotional wellbeing including fear of more horizontal violence and more psychological harm.

Experiencing threats to one's well-being and fear of future horizontal violence interferes with communication. DeVito [33] illustrates communication between people and the factors that promote or impede it in his essentials of human communication model. He defines communication as the interpersonal exchange of verbal and nonverbal messages between people [33]. He explains that, at one extreme, a sender's message will not reach an intended recipient at all because of psychological noise, a factor that impedes communication. Psychological noise includes thoughts about or beliefs and attitudes formed in advance of the communication and/or strong negative feelings about how that communication may occur.

In the proposed model displayed in Figure 1, safety needs and psychological noise provide the link between horizontal violence and peer communication; nurses who have experienced horizontal violence may avoid interacting with their peers because of perceived threats to psychological wellbeing and preconceived notions about how the communication exchange will play out. Using safety needs and psychological noise to link them, the proposition is that horizontal violence is negatively related to peer communication; that is, as horizontal violence increases, peer communication decreases.

### 2.4. Quality of Care

Quality of care is the extent to which care delivered to patients increases the chance of meeting their needs [45]. Good quality of care is culturally sensitive and clearly communicated care that is delivered competently while involving the patient in decisions about their care. Further, the first dimension of high-quality care is that it is safe and does not result in injury to patients [45].

### 2.5. Peer Communication and Quality and Safety of Patient Care

Reason's [34] Swiss cheese model of system accidents illustrates how people and things get harmed in technologically sophisticated organizations including healthcare. He developed the model to promote evaluation of bad outcomes by considering what failed in a system's defense layers rather than simply blaming people for the errors. In his conceptualization, these layers protect people and things from harm. They consist of people, such as nurses and pilots, technology such as alarms, and policies and procedures that each play a vital part and, collectively, are usually protective. Conversely, when these layers are compromised, an opportunity for an error to cause harm exists. The defense layer of interest is the one comprised of people that, in healthcare, consists of those caring directly for patients including their communication with each other. Without open communication among caregivers or the people in the defense layer, the potential for detecting and preventing harm is reduced.

In the proposed model displayed in Figure 1, peer communication is hypothesized as one of many important contributors to protecting patients from harm. Communication among nurses is conceptualized as sharing information related to the care of patients including asking each other questions, providing feedback to each other, giving each other advice or seeking clarification, or validation of care. Decreased peer communication is hypothesized to threaten the integrity of the defense layer.

The concept of the quality and safety of patient care is used to name the process of delivering care that meets the needs of an individual where patient harm is evaded and averted [45, 46]. In the model, using defense layers to link them, peer communication is positively related to the quality and safety of patient care, that is, as peer communication decreases, so does the quality and safety of patient care.

## 3. Implications for Research

The horizontal violence and the quality and safety of patient care model offers a framework to guide research where there is a paucity of empirical evidence on a topic of growing concern among nurses internationally. The model and its propositions generate research hypotheses for testing. Hypothesis one: the model suggests that as internalized dominant values exhibited as oppressed group or self increase, so does horizontal violence. Hypothesis two: the model suggests as horizontal violence increases, peers communication decreases. Hypothesis three, the model suggests that as peer communication decreases, so does the quality and safety of patient care. Mounting evidence of empirical links, or lack thereof, validates and provides opportunity for improvement of the model [36, 47]. Evidence of empirical links creates a new call for research to inform strategies for addressing horizontal violence and its consequences for patients.

## 4. Conclusion

This paper presented the horizontal violence and the quality and safety of care model. Four theories linked for the first time illustrate how horizontal violence arises and its effect on the quality and safety of patient care. Internationally, nurses share concern about horizontal violence and its consequences. Studies suggest that nurses suffer consequences as a result of their experiences with horizontal violence; yet little, if anything, is known about consequences for patients and no known framework exists to explain or guide research on the topic. The new model begins to fill this gap. However, research is needed to validate the new model. Empirical evidence gathered from studies guided by the model will establish the foundation of practice and education recommendations.

## References

[1] Blanton BA, Lybecker C, Spring NM A horizontal violence position statement. http://proactivenurse.com/index.php?option=com_content&Itemid=22&id=83.

[2] Farrell GA (1997). Aggression in clinical settings: nurses’ views. *Journal of Advanced Nursing*.

[3] Farrell GA (1999). Aggression in clinical settings: nurses’ views—a follow-up study. *Journal of Advanced Nursing*.

[4] Hughes RG, Clancy CM (2009). Complexity, bullying, and stress: analyzing and mitigating a challenging work environment for nurses. *Journal of Nursing Care Quality*.

[5] Johnson SL, Rea RE (2009). Workplace bullying: concerns for nurse leaders. *Journal of Nursing Administration*.

[6] Simons S (2008). Workplace bullying experienced by massachusetts registered nurses and the relationship to intention to leave the organization. *Advances in Nursing Science*.

[7] Randle J (2003). Bullying in the nursing profession. *Journal of Advanced Nursing*.

[8] Vessey JA, DeMarco RF, Gaffney DA, Budin WC (2009). Bullying of staff registered nurses in the workplace: a preliminary study for developing personal and organizational strategies for the transformation of hostile to healthy workplace environments. *Journal of Professional Nursing*.

[9] Cox H (1991). Verbal abuse nationwide, part I: oppressed group behavior. *Nursing Management*.

[10] Rowe MM, Sherlock H (2005). Stress and verbal abuse in nursing: do burned out nurses eat their young?. *Journal of Nursing Management*.

[11] Sofield L, Salmond SW (2003). Workplace violence. A focus on verbal abuse and intent to leave the organization. *Orthopaedic Nursing*.

[12] Griffin M (2004). Teaching cognitive rehearsal as a shield for lateral violence: an intervention for newly licensed nurses. *Journal of Continuing Education in Nursing*.

[13] Sheridan-Leos N (2008). Understanding lateral violence in nursing. *Clinical Journal of Oncology Nursing*.

[14] Stanley KM, Martin MM, Michel Y, Welton JM, Nemeth LS (2007). Examining lateral violence in the nursing workforce. *Issues in Mental Health Nursing*.

[15] Felblinger DM (2008). Incivility and bullying in the workplace and nurses’ shame responses. *Journal of Obstetric, Gynecologic, and Neonatal Nursing*.

[16] Thomas SP (2003). Horizontal hostility nurses against themselves: how to resolve this threat to retention. *The American Journal of Nursing*.

[17] Alspach G (2007). Critical care nurses as coworkers: are our interactions nice or nasty?. *Critical Care Nurse*.

[18] McKenna BG, Smith NA, Poole SJ, Coverdale JH (2003). Horizontal violence: experiences of registered nurses in their first year of practice. *Journal of Advanced Nursing*.

[19] Quine L (2001). Workplace bullying in nurses. *Journal of Health Psychology*.

[20] Georgiou G (2007). Anti-bullying tactics make a difference. *RCM Midwives*.

[21] Moye M Nursing hostility: what is causing horizontal violence between nurses and what steps can individuals take to bring it to an end? Advance for nurses. http://nursing.advanceweb.com/Editorial/Content/PrintFriendly.aspx?CC=214570.

[22] Stewart S (2010). Confronting bullying. *Nursing New Zealand*.

[23] Dunn H (2003). Horizontal violence among nurses in the operating room. *AORN journal*.

[24] Skillings LN, Thompson JL, Allen DD, Rodrigues-Fisher L (1992). Perceptions and feelings of nurses about horizontal violence as an expression of oppressed group behavior. *Critique, Resistance, and Action*.

[25] Rosenstein AH, O’Daniel M (2005). Disruptive behavior and clinical outcomes: perceptions of nurses and physicians. *The American Journal of Nursing*.

[26] Rosenstein AH, O’Daniel M (2008). A survey of the impact of disruptive behaviors and communication defects on patient safety. *Joint Commission Journal on Quality and Patient Safety*.

[27] Institute for Safe Medication Practices https://www.ismp.org/survey/surveyresults/survey0311.asp.

[28] The Joint Commission Sentinel event data: root causes by event type 2004-third quarter2011. http://www.jointcommission.org/assets/1/18/Root_Causes_Event_Type_2004-3Q2011.pdf.

[29] Freire P (2003). *Pedagogy of the Oppressed*.

[30] Longo J (2007). Horizontal violence among nursing students. *Archives of Psychiatric Nursing*.

[31] Meleis AI (2007). *Theoretical Nursing Development & Progress*.

[32] Maslow AH (1943). A theory of human motivation. *Psychological Review*.

[33] DeVito JA (2008). *Essentials of Human Communication*.

[34] Reason J (2000). Human error: models and management. *British Medical Journal*.

[35] Earp JA, Ennett ST (1991). Conceptual models for health education research and practice. *Health Education Research*.

[36] Purpora C, Blegen MA, Stotts NA Horizontal violence between hospital staff nurses related to oppressed self or oppressed group.

[37] Roberts SJ (1983). Oppressed group behavior: implications for nursing. *Advances in Nursing Science*.

[38] Ashley J (1976). *Hospitals, Paternalism, and the Role of the Nurse*.

[39] Reverby SM (1987). *Ordered to Care: The Dilemma of American Nursing*.

[40] Cicchetti A (1986). Networks: between markets and hierarchies. *Strategic Management Journal*.

[41] Garman AN, Leach DC, Spector N (2006). Worldviews in collision: conflict and collaboration across professional lines. *Journal of Organizational Behavior*.

[42] DeMarco R, Roberts SJ, Norris A, McCurry MK (2008). The development of the nurse workplace scale: self-advocating behaviors and beliefs in the professional workplace. *Journal of Professional Nursing*.

[43] Fanon F (1963). *The Wretched of the Earth*.

[44] Keen P, Neil RM, Watts R (1991). Caring for ourselves. *Caring and Nursing: Exploration in Feminist Perspectives*.

[45] The Institute of Medicine (2001). *Crossing the Quality Chasm: A New Health System for the 21st Century*.

[46] Agency for Healthcare Research and Quality http://www.ahrq.gov/qual/patientsafetyculture/hospscanform.pdf.

[47] Purpora C (2010). *Horizontal Violence among Hospital Staff Nurses and the Quality and Safety of Patient Care*.

